# Raccoon Social Networks and the Potential for Disease Transmission

**DOI:** 10.1371/journal.pone.0075830

**Published:** 2013-10-10

**Authors:** Ben T. Hirsch, Suzanne Prange, Stephanie A. Hauver, Stanley D. Gehrt

**Affiliations:** 1 School of Environment and Natural Resources, The Ohio State University, Columbus, Ohio, United States of America; 2 Smithsonian Tropical Research Institute (STRI), Balboa, Panama; 3 Ohio Division of Wildlife, Athens, Ohio, United States of America; 4 School of Education, Binghamton University, Binghamton, New York, United States of America; Centers for Disease Control and Prevention, United States of America

## Abstract

Raccoons are an important vector of rabies and other pathogens. The degree to which these pathogens can spread through a raccoon population should be closely linked to association rates between individual raccoons. Most studies of raccoon sociality have found patterns consistent with low levels of social connectivity within populations, thus the likelihood of direct pathogen transmission between raccoons is theoretically low. We used proximity detecting collars and social network metrics to calculate the degree of social connectivity in an urban raccoon population for purposes of estimating potential pathogen spread. In contrast to previous assumptions, raccoon social association networks were highly connected, and all individuals were connected to one large social network during 15 out of 18 months of study. However, these metrics may overestimate the potential for a pathogen to spread through a population, as many of the social connections were based on relatively short contact periods. To more closely reflect varying probabilities of pathogen spread, we censored the raccoon social networks based on the total amount of time spent in close proximity between two individuals per month. As this time criteria for censoring the social networks increased from one to thirty minutes, corresponding measures of network connectivity declined. These findings demonstrate that raccoon populations are much more tightly connected than would have been predicted based on previous studies, but also point out that additional research is needed to calculate more precise transmission probabilities by infected individuals, and determine how disease infection changes normal social behaviors.

## Introduction

Raccoons are a common species throughout North America, as well as host to a wide range of pathogens. Some of these are important zoonotic diseases or diseases of domestic animals, such as rabies, canine distemper, parvovirus, leptospirosis, etc. [1]. Among these diseases, rabies is perhaps the most important from a human health perspective, and understanding how pathogens are transmitted in raccoon populations is important for devising effective management and disease abatement strategies. Because the spread of rabies in wild raccoon populations has been well documented across much of the US (e.g. [2]), rabies incidents are a particularly useful model for studying and understanding the spread of pathogens in wild raccoons. However, despite the wealth of documented rabies cases, very little is known about patterns of pathogen transmission between individual raccoons. For this reason, it is important to understand the structure of association patterns in wild raccoons and how these associative behaviors could affect pathogen transmission [3]–[5].

Rabies infection results in viral encephalomyelitis and leads to thousands of human deaths annually [6]. It is believed that raccoons are typically infected after being bitten by a rabid animal. The rabies virus then spreads through the nervous system and the brain, multiplies rapidly, and passes to the salivary glands [7]. During the initial incubation period (which averages approximately 3–12 weeks), raccoons show no sign of the disease [8]–[9]. Once an animal develops signs of the disease, they normally die within seven days, and the disease is almost always fatal [8], [10]–[11]. Raccoons are able to infect others for a short period before they die, and the probable infection window is on the order of one week [11].

An epizootic variant of raccoon rabies was first documented in Florida during the 1940′s and was introduced to the West Virginia/Virginia border during the 1970′s [12]–[14]. This raccoon rabies variant proceeded to spread across most of the Northeast, as well as into Canada [10]. One puzzling aspect of the recent rabies epizootic in raccoons is that it has spread so quickly, at a rate of 30–47 km/year [15]–[16], and has resulted in more than 50,000 reported cases of raccoon rabies since 1980 [10]. Long distance dispersal by young male raccoons and translocations of infected individuals were likely major factors driving this rapid spread of rabies [17]–[20], although it is highly likely that characteristics of local raccoon populations were also important [21].

Although local population density has been factored into some rabies models [22], little is known concerning the detailed patterns of social interactions between raccoons (but see: [3], [5], [23]). These population wide association patterns, or social networks, are crucial for understanding the epidemiology of raccoon pathogen transmission [24]–[26]. Traditional SIR (susceptible-infectious-recovered) disease models assume population wide random-mixing of individuals, but most wild animals do not associate randomly. Because deviations from random assortment can greatly affect disease transmission models, some knowledge of the basic social network structure is needed to compute more accurate dynamic models based on individual-level behavior [25]. If raccoon social networks exhibit particularly high connectivity and individuals frequently associate with each other, this could help explain why rabies has spread so rapidly and provide vital information for preventing further outbreaks.

Raccoons have often been considered solitary [27]–[31], which is not conducive to the rapid spread of pathogens. However, several recent studies have found that raccoon sociality is more flexible than previously thought, and that males often form social groups whose behaviors include traveling, foraging, and denning together [32]–[35]. In particular, some studies have shown that groups of 2–4 adult males share home ranges exclusive of other males [35]. Adult female home ranges typically overlap with adult males, but adult males and females in most studies have not been reported to interact frequently outside the mating season (but see: [5], [23], [36]). If adult males are spatially segregated, the raccoon social network should not be highly connected unless males regularly interact with neighboring males and/or intrasexual encounters are common. In some populations, the density of raccoons is so high that males are unable to defend territories [37]–[39] and this system of overlapping male home ranges may allow frequent transfer of pathogens through adult males. Although adult females are less social than males, they frequently associate with juvenile offspring, and mother-offspring transmission is hypothesized to be a common rabies infection pathway [18]. Even after factoring in mother-offspring interactions, most studies of raccoons sociality do not report large numbers of conspecific interactions, thus raccoon social networks are not predicted to be particularly well connected.

The goal of this paper is to use patterns of raccoon contact rates described in [5], [36] to explore the relationship between social structure and the potential for pathogen transmission. We used proximity logging technology to record the amount of time adult raccoons spent in close proximity [39]. These proximity logging collars also allow us to document and quantify brief or infrequent interactions that are difficult to detect using traditional radio-tracking [5], [39]–[40]. Through the use of this technology, we constructed detailed social proximity metrics, which are crucial for understanding pathogen spread within a wildlife population (e.g. [41]–[42]). We hypothesized that the rapid spread of raccoon rabies, and other directly transmissible pathogens, may be facilitated by highly connected raccoon social networks, which are ideal for transmitting pathogens.

## Methods

### Ethics statement

This study conformed to ASAB/ABS guidelines for animal welfare. Animal trapping and handling procedures were approved by the Ohio State University Institutional Animal Use and Care Committee (IACUC#2003R0062).

### Study area

Fieldwork was conducted within a 20-ha portion of the 1,499-ha Ned Brown Forest Preserve in suburban Cook County, Illinois (for further details of the site, see: [5], [37], [38]). Permission for working in the Ned Brown Forest Preserve was given by the Forest Preserve District of Cook County. The size of the study area was determined by the local density of raccoons, as it was important to monitor all, or nearly all, resident raccoons [5]. The high density of raccoons at this site (40–70 raccoons/km^2^) was likely related to the abundance of artificial food sources available from garbage cans [37]–[38]. Raccoons generally breed once per year, and average litter sizes from the study area are 3.56 per year (range 2–6; Stanley Gehrt unpublished data). Home range sizes average 38 ha (range 10.7–325.6), with males having slightly larger home ranges [38]. From May 2004 to Dec 2005, raccoons were trapped in box traps, immobilized with an injection of Telazol (as in: [43]), weighed, sexed, and individually tagged. All raccoons >12 months of age were fitted with proximity logging radio-collars (SirTrack Ltd., Havelock North, New Zealand) which recorded the identity and length of contact when two radio-tracked raccoons were within 1–1.5 m proximity (for details see: [39]). Raccoons were aged according to tooth wear [44]. We condensed age classes of collared adult raccoons into two categories: young adults (12–38 months) or old adults (≥39 months) following [5]. A total of 42 adults (20 males and 22 females) were collared, and these individuals represented close to 100% of all adult raccoons living in the core area [5]. Raccoons in this population were re-trapped repeatedly to replace malfunctioning collars and to maintain a high population of marked individuals in the population.

### Statistical methods

We analyzed data from a total of 30 raccoons (11 male, 19 female) in our study population and the monthly social network matrices contained an average of 16.2 individuals per month (range 10–24). The number of raccoons with contact data decreased over time because animals died (N = 4) and proximity collars expired. Because individuals with properly functioning proximity collars entered and exited our study over time, creating social networks over long time periods (3 months or more) was problematic. We instead partitioned the association data into one month increments, which allowed us to maximize the number of individuals included in the analyses. Any individuals that died, or were without a functioning radio-collar for >10 days during a particular month were censored from the monthly association matrix. Association matrices were constructed using the total amount of time two individuals spent in close proximity (within 1–1.5 m) during that month divided by the total number of days in the month.

Social network metrics were calculated for all individuals in our networks. In particular, we focused on individual network measures that are related to disease transmission including: 1) weighted degree (the proportion of individuals in a group or population that were observed to associate with an individual), 2) two step reach (the proportion of individuals that one individual is connected to within two steps), and 3) clustering coefficient (which is a measure of the degree to which well connected individuals are connected to other well connected individuals). Group level measures were calculated for each of the 18 months in our study. We used measures of network density (the total number of associations divided by the total number of possible associations) and connectedness (the proportion of pairwise associations that are contained within the largest network component) to give general estimates of how easily a pathogen could infect the individuals in our population. If all individuals associated with all other individuals in the population, the network density value would equal one. Even if the network density is less than one, all individuals in the population may be connected through associations with other individuals, in which case the network connectivity value would equal one.

Many social network statistics do not differentiate between strong and weak connections. In our dataset, an individual's degree (the number of associates) could include pairs of individuals that were only associated for a total of two seconds over a one-month period. Given that the infection window of rabies is approximately one week [11], social networks calculated from all association data may overestimate the degree of connectivity with respect to the transmission of rabies and some other pathogens. To address this issue, we subsampled our dataset using different censoring criteria to investigate varying levels of network connectivity in our raccoon population based on different assumptions concerning the probability of pathogen transmission. We re-calculated the previously described social networks statistics using censored networks where dyads were only considered connected if they associated for a minimum of 1, 5, 15, or 30 minutes during the month. Although it is unknown how many interactions, or how long raccoons need to associate to successfully transmit rabies and other pathogens, the use of four different criteria for censoring the social networks allows us to make general conclusions about the potential for pathogen transmission through raccoon social networks given differing contact rates. The resultant patterns derived from these different censoring criteria may be analogous to modeling diseases with different transmission rates. Non-censored networks may represent possible infection pathways for easily transmittable pathogens, while strict time-censored networks could indicate transmission pathways for diseases with low transmission rates.

## Results

**Figure 1:**
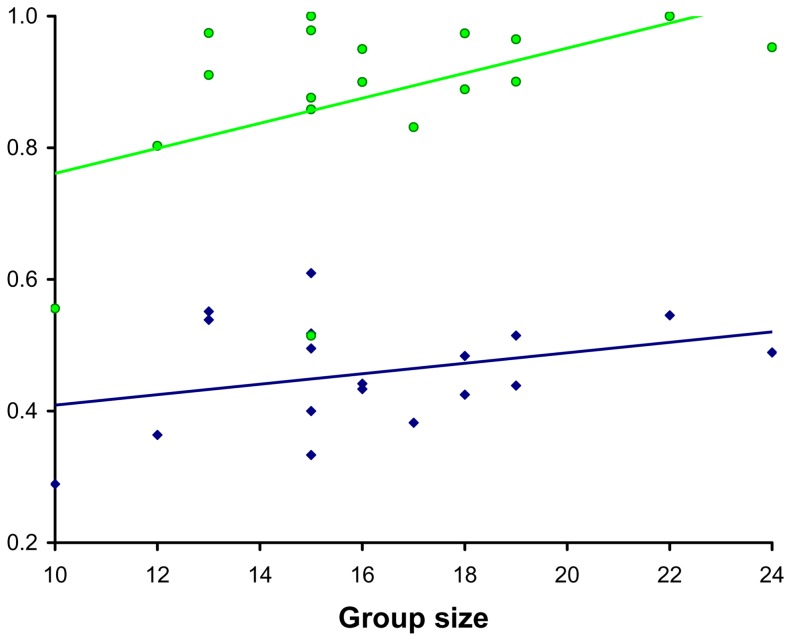
Average normalized degree (blue triangles), and two-step reach (green circles) in relation to network size. Average normalized degree values were not significantly correlated (R^2^ = 0.106, F_1,18_ = 1.893, P = 0.188), while two-step reach values were significantly higher (R^2^ = 0.225, F_1,18_ = 4.655, P = 0.047).

Monthly social association networks were highly connected (Table 1). The average number of individuals per network was 16.22±3.44 SD, and raccoon individuals associated with an average of 7.07±2.24 SD other individuals per month, resulting in an average normalized degree of 0.46±0.08 SD. The two-step reach values were notably higher, averaging 0.88±0.14 SD. Overall levels of connectedness in the social networks were remarkably high (monthly average  = 0.95±0.12 SD), and 15 of the 18 monthly social networks had connectedness values equal to 1 (i.e. every individual in the population was connected to one large social network). In general, our social network measures were not closely correlated to social network size (logistic regression p values >0.05). Average normalized degree values were slightly higher during months with larger social network size (R^2^ = 0.106, F_1,18_ = 1.893, p = 0.188), and two step reach values were significantly higher (R^2^ = 0.225, F_1,18_ = 4.655, p = 0.047) indicating the removal of individuals from the population does lead to some decrease in network connectivity (Figure 1). Even though individuals spend more time in close proximity and contacted each other more frequently during the winter [5], which overlaps with the December-March mating season, social networks during these months were not more compact (average compactness April-November  = 0.721, range  = 0.624–0.805; December-March  = 0.616, range  = 0.444–0.771).

**Table 1:** Monthly network metrics.

Network measure	July	Aug	Sept	Oct	Nov	Dec	Jan	Feb	Mar	April	May	June	July	Aug	Sept	Oct	Nov	Dec	Aver age		
**Network N**	24	22	19	18	19	15	15	13	15	18	17	16	15	16	15	13	12	10	16.222	±	3.440
**Average Degree**	11.250	11.455	9.263	8.222	7.895	7.250	4.667	6.615	5.600	7.222	6.118	6.500	6.933	6.625	8.533	6.462	4.000	2.600	7.067	±	2.237
**Normalized degree**	0.489	0.545	0.515	0.484	0.439	0.518	0.333	0.551	0.400	0.425	0.382	0.433	0.495	0.442	0.610	0.539	0.364	0.289	0.458	±	0.084
**2 step reach**	0.952	1.000	0.965	0.974	0.900	0.858	0.514	0.974	0.876	0.889	0.831	0.950	0.978	0.900	1.000	0.910	0.803	0.556	0.880	±	0.138
**Connectedness**	1.000	1.000	1.000	1.000	1.000	1.000	0.657	1.000	1.000	1.000	0.882	1.000	1.000	1.000	1.000	1.000	1.000	0.622	0.953	±	0.118
**Compactness**	0.741	0.773	0.751	0.737	0.701	0.717	0.467	0.771	0.679	0.694	0.624	0.708	0.740	0.704	0.805	0.754	0.645	0.444	0.692	±	0.097
**Clustering coefficient**	0.702	0.780	0.722	0.677	0.699	0.640	0.669	0.675	0.688	0.810	0.685	0.686	0.684	0.670	0.597	0.656	0.714	0.734	0.694	±	0.048
**Betweenness**	0.162	0.180	0.152	0.242	0.216	0.275	0.257	0.449	0.238	0.196	0.140	0.204	0.083	0.204	0.224	0.224	0.197	0.233	0.215	±	0.074

**Figure 2:**
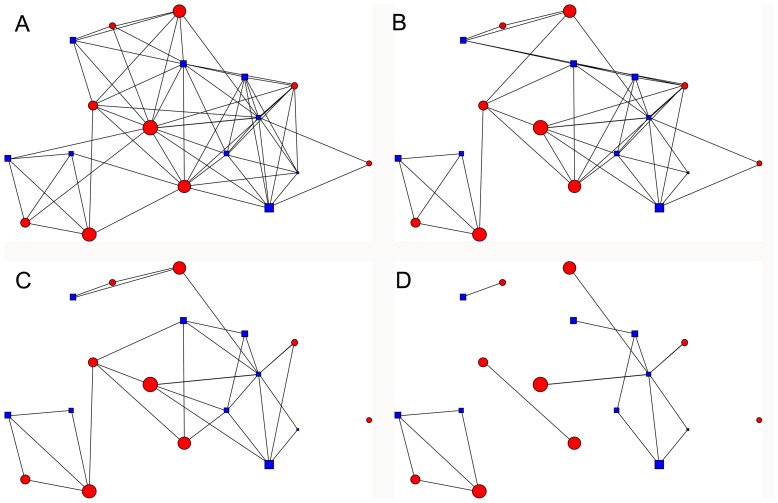
Time censored raccoon association networks (October 2004). Red circles  =  females, blue squares  =  males. Symbol size is scaled to age, with older individuals having larger symbols. Associations between individuals are represented with a line. Each association network is constructed based on the total time spent in proximity per month between individuals: A. monthly dyadic contact time ≥1 min., B. ≥5 min. C. ≥15 min. & D. ≥30 min.

**Figure 3:**
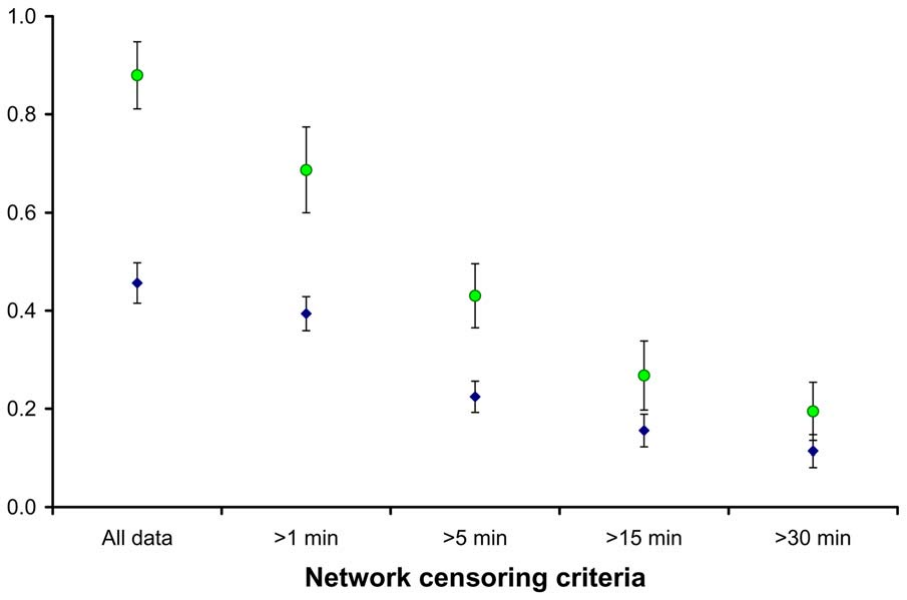
Average normalized degree (blue triangles), and two-step reach (green circles) in relation to the degree of time censoring of the social networks. Error bars indicate the 95% confidence intervals.

**Figure 4:**
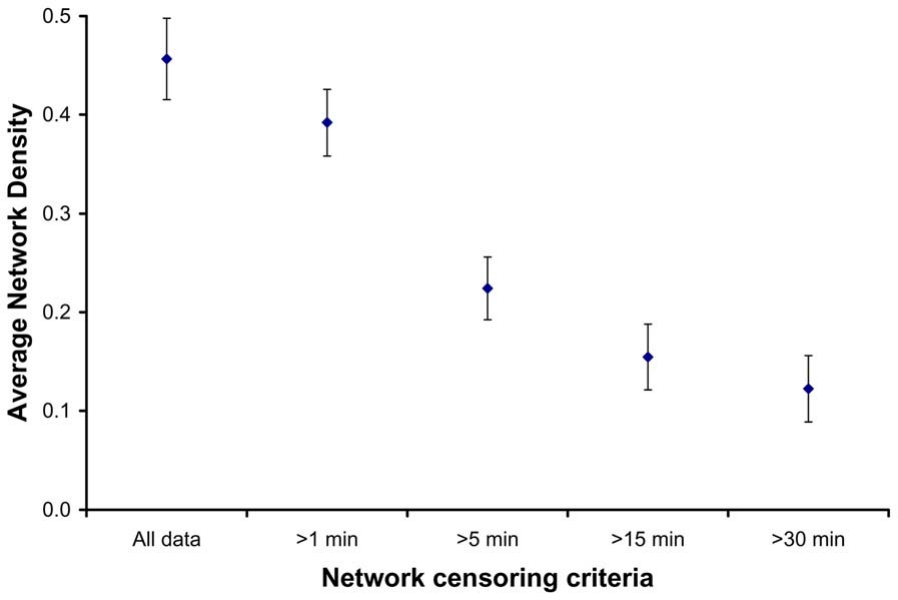
Average network connectivity in relation to the degree of time censoring. Error bars indicate the 95% confidence intervals.

**Figure 5:**
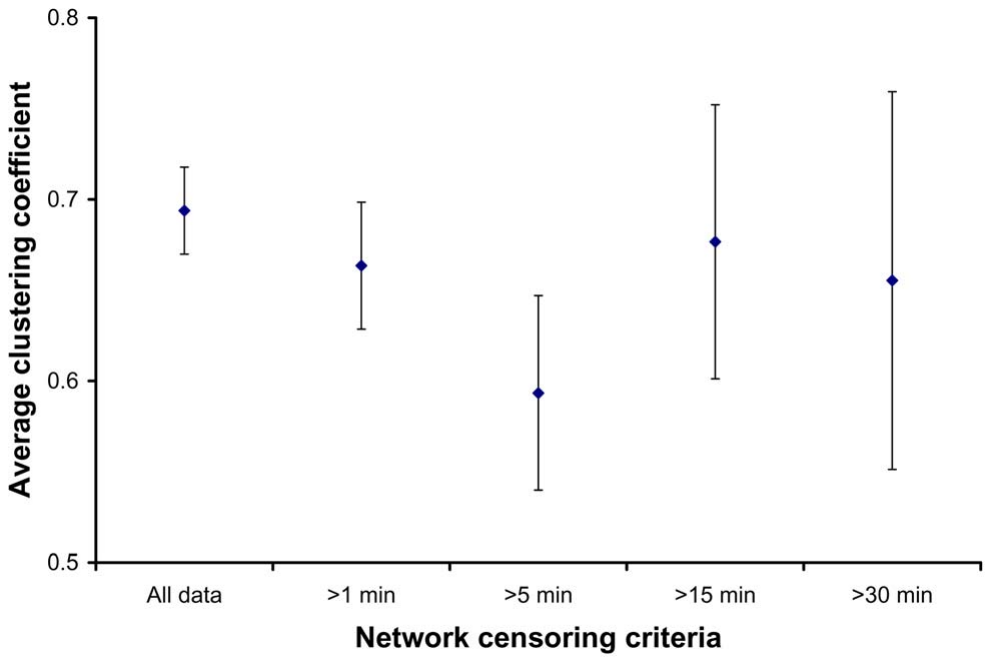
Average network clustering coefficients in relation to the degree of time censoring. Error bars indicate the 95% confidence intervals.

The time censored networks were not as well connected as the full social networks (Figure 1a in [36], Figure 2). Both average normalized degree and two step reach declined as the time censoring criteria became more stringent (from 1 to 30 minutes; Figure 3), and many of the social connections between raccoon dyads were based on relatively infrequent and quick associations (34% of all dyadic associations consisted of <1 minute per month). Dyadic interactions that summed to thirty minutes or more over the course of a month made up a small percentage (19.12%) of dyadic associations, thus censoring all shorter associations from our social networks logically led to lower connectivity. For example, in the full social networks, connectedness averaged 0.95±0.12 SD, and plummeted to 0.23±0.19 SD when we only included dyads that interacted thirty minutes or more per month (Figure 4). Although clustering has been shown to have a major effect on the growth rate of epidemics [45], there was no clear linear relationship between time censoring and clustering coefficient values (Figure 5), which could have been related to the large number of missing clustering coefficient values from individuals not connected to the censored networks.

## Discussion

The full monthly raccoon social networks (e.g. without data censoring) were all highly connected (Table 1, [36]). In general, the raccoons in this study population were part of one large social network, and all individuals were connected to the social network during 15 out of 18 months. This is similar to patterns found in Tasmanian devil (*Sarcophilus harrisii*) social networks, where the transmission of facial tumors has spread through the population and is a major threat to the species [41], [46]. Measures of average normalized degree, network density, and two step reach all indicated our raccoon social networks were highly connected. Individual raccoons in our population associated with almost 50% of other individuals within the study area (as measured by average normalized degree). This high level of social connectivity has not been recorded in previous studies of raccoons using radio-telemetry [32]–[35]. Although these studies have found high rates of male-male associations, traditional studies have not reported frequent mixed-sex associations outside of the mating season. Such sexual segregation should result in highly disconnected social networks that are not conducive to pathogen transmission. Although female-female and male-female associations outside the mating season were generally less common and shorter in duration than male-male associations, they were still quite common in our study population [5]. This lack of sexual segregation was a primary factor leading to high levels of social network connectivity.

The high levels of connectivity in the Ned Brown Forest Preserve social networks may be influenced by the high density of raccoons in this urban population. While our population may not be representative of all raccoon populations, we posit that the social networks reported here could be similar to other urban raccoon populations. Previous work has shown that raccoons with a more concentrated distribution of food resources spread diseases more easily [4]. In addition, understanding pathogen transmission dynamics in these dense urban raccoon populations may be particularly important for preventing pathogen spillover into human populations. One potential method of addressing the issue of population density variance is to compare networks of different size within the study population. The total size of these raccoon social networks declined from 24 individuals during July 2004 to 10 individuals during December 2005, mostly as a result of collar failure. These collar failures could be analogous to artificially removing individuals from an existing social network. When group size is regressed against normalized one- and two-step reach both values decline as group size decreases, although the effect is only significant for two-step reach (Figure 2). This result demonstrates that lower density raccoon populations should have lower network connectivity than high density populations. Although the patterns were consistent with this predicted effect, the degree to which network size affected one and two-step reach was relatively weak. This result may indicate that when infected individuals die, the subsequent social networks may not lose a great deal of their connectivity. These results may also be applicable to raccoons living in lower density populations (also see [47]). We posit that further studies of raccoon social network connectivity should be conducted across a broad range of population densities to better address the effect of population density on the potential for disease transfer.

Because the social networks were highly connected, it could be concluded that rabies infections should rapidly sweep through this population. On the other hand, very little is known about the probability of pathogen transmission between adult raccoons. While rabies is typically transmitted via bites [8], it is unknown how frequently raccoons bite each other. Video monitoring in our study area revealed that raccoons interacted aggressively during only 7% of interactions occurring at feeding stations (Hauver et al. unpublished data). Given that aggression typically increases at concentrated food resources [48]–[49], the average amount of aggression and social contacts between healthy raccoons in non-human-altered habitats may be much lower [50]. Although raccoons infected with rabies may be more aggressive than uninfected individuals, it is likely that tenuous connections, such as brief encounters at feeding sites, may not be generally sufficient for the transmission of rabies. Given the unpredictability of aggression between associated raccoons, and the roughly one week rabies infection window, we posit that the time censored networks are probably a better representation for the potential for rabies transfer between raccoons. Alternately, pathogens with longer infectious periods, and easier pathways for transmission (such as airborne or droplet transmission) may be most appropriately modeled using the non-censored networks.

All social network measures calculated here declined as the time censoring criteria increased from zero to thirty minutes (with the exception of clustering coefficients). This pattern indicates that raccoon disease transfer networks are probably not as connected as the complete social proximity networks. When comparing the complete networks versus the one-minute censored networks, total network connectivity and average normalized degree changed very little (Figures 3 & 4), but dropped considerably when networks were censored at fifteen minutes or more. This leads us to conclude that if a raccoon pathogen is easily transmitted during brief, infrequent contacts between individual raccoons, the social networks we describe here are ideal for disease transmission. Alternately, if pathogen transmission most commonly occurs between individuals who spend at least fifteen minutes together over the course of a month, the complete raccoon social networks are probably not adequate representations of disease transmission networks. The close social associations in this population are driven, in part, by co-denning behaviors, as demonstrated by increases in dyadic association times during cold winter months [5], [23]. Given that co-denning individuals likely groom and lick each other, we surmise that the possibility of infection is much higher for co-denning individuals than raccoons meeting briefly at food resources. While the exact behavior of co-denning animals is unknown, our conclusions about disease transmission pathways are partially supported by the infrequent occurrence of biting, licking, and grooming at video monitored feeding stations (Hauver et al. unpublished data [51]).

Additional mechanisms that are not represented in our social networks could potentially play a large role in the dissemination of raccoon rabies. In some cases, rabid raccoons have been observed to behave particularly aggressively [18], thus rabies may lead to behavioral changes which increase the amount of bites to conspecifics compared to patterns seen in non-infected individuals. This in turn could lead to higher rates of pathogen transfer. Alternately, some infected raccoons act sick, have uncoordinated movements, and become effectively paralyzed [18], [52]–[54]. Given the wide variation in behaviors exhibited by rabid raccoons, it is difficult to predict exactly how these behavioral changes affect infection patterns, and it would be ideal to have a greater knowledge of the association behaviors of infected individuals. The transmission of pathogens can also occur through contagion pathways not represented in our data. The social networks here constitute contacts between adult raccoons, and do not include young juveniles that are dependent on their mothers (these individuals are too small for radio-collars). Rosatte [18] stated that “the potential for transmission of rabies is great between the adult female raccoon and her young.” In this same study, juvenile rabies infections peaked in early fall, which corresponded to mother-offspring rabies transmission [18]. Alternately, population level temporal peaks in rabies abundance appear to be tightly linked to the mating season or an increase in communal denning during winter months, which could also be consistent with adult-adult pathogen transfer [18], [53], [55]–[56]. These studies suggest that there are multiple pathways to rabies transmission in raccoons: 1) mother to offspring, 2) adult male to adult male, and 3) between mating adult males and females. In contrast, our results also demonstrate the likely presence of two additional infection pathways: 1) between adult males and females outside the mating season, and 2) between adult females.

In our study population, positive associations between pairs of adult males (as measured by the total amount of time spent together) were relatively stable over time, and these preferred associations frequently lasted through multiple seasons [5]. On the other hand, positive associations among adult females were less stable over time, and involved less time spent in close proximity than male-male associations [5]. The seasonal changes in close association partners, particularly during the mating season, could lead to much higher levels of connectedness over time than observed in our monthly static network models. This could also explain how pathogens are able to spread so quickly through raccoon populations, even if they are fairly difficult to transmit. Indeed, the next step in understanding the role of sociality in relation to the spread raccoon pathogens is to use dynamic social networking tools to model pathogen spread using observed association data.

The social proximity networks presented here can shed light on the transmission of many important diseases such as the canine distemper virus (CDV), parvovirus, and leptospirosis [57]–[58]. Given that different pathogens have different methods of transmission, the ability for these pathogens to spread through the raccoon social network should be different. For example, CDV is transmitted through the aerosolization of respiratory exudates [59]. This infection pathway is likely to be more closely reflected through our use of proximity logging collars than rabies, which typically is transmitted through biting. Given the highly connected social networks reported here and the much longer infection window for CDV transmission (60–90 days; [60]), urbanized raccoon populations are likely excellent reservoirs for CDV. Indeed, multiple studies have found that CDV is endemic in raccoon populations [58], [61]–[64], and that these populations are important reservoirs for CDV spillover into domestic dogs and zoo animals [59]. We posit that a better understanding of infection pathways and transmission probabilities, in combination with detailed descriptions of social contact networks, are an important step for understanding the epidemiology of wildlife pathogens.

Raccoon social patterns in our study population are much more complex and extensive than previously reported. Our study demonstrates that raccoons contact a substantial proportion of individuals within their population, and that their social proximity networks are highly connected. While this result has important implications for the transmission of rabies and other pathogens, there are still several important factors that can influence the epidemiology of raccoon pathogens. As previously mentioned, more information is needed about how social and aggressive behavior change when raccoons are infected with rabies. Additionally, if a disease sweeps through a population and a large proportion of the raccoons die, how does this affect the social proximity network? If raccoons do not change their normal social associations and movement patterns, social network connectivity and pathogen transmission should decline. However, if surviving raccoons seek out new social partners, the likelihood of pathogen spread through a population may remain high, even after an outbreak. We suggest that future studies should closely monitor the behavior and social interactions of raccoons before, during, and after disease outbreak. In addition, given that concentrated anthropogenic food sources can lead to an increase in raccoon associations [50], [65], measures aimed at reducing the amount of exposed trash bins and concentrated food items available to raccoons may be an important management tool for reducing pathogen transmission. Not only could this strategy reduce the number of raccoon social contacts, but it may reduce raccoon numbers in high density populations that heavily depend on human sourced foods.

## References

[1] Gehrt SD (2003) Raccoons and allies. In: Feldhamer, GA, Chapman JA, Thompson BC editors. Wild Mammals of North America, 2^nd^ edition. Baltimore: Johns Hopkins University Press. pp 611–634.

[2] Blanton JD, Plamer D, Dyer J, Rupprecht CE (2011) Rabies surveillance in the United States during 2010. JAVMA, 239, 773–783. 10.2460/javma.239.6.773 PMC5120392 21916759

[3] Totton S, Tinline R, Rosatte R, Bigler L (2002) Contact rates of raccoons (Procyon lotor) at a communal feeding site in rural eastern Ontario. Journal of Wildlife Diseases. 38: 313–320. 10.7589/0090-3558-38.2.313 12038131

[4] Gompper ME, Wright AN (2005) Altered prevalence of raccoon roundworm (*Baylisascaris procyonis*) owing to manipulated contact rates of hosts. Journal of Zoology 266: 215–219.

[5] Prange S, Gehrt SD, Hauver S (2011) Frequency and duration of contacts between free-ranging raccoons: uncovering a hidden social system. Journal of Mammalogy 92: 1331–1342.

[6] Meltzer MI, Rupprecht CE (1998) A review of the economics of the prevention and control of rabies: Part1: Global impact and rabies in humans. Pharmacoeconomics 14: 365–383. 10344905 10.2165/00019053-199814040-00004

[7] Rupprecht CE, Hanlon CA, Hemachudha T (2002) Rabies re-examined. Lancet Infectious Diseases 2: 327–343. 12144896 10.1016/s1473-3099(02)00287-6

[8] McLean RG (1975) Raccoon rabies. In: Baer GM editor. The natural history of rabies. New York: Academic Press. pp. 53–76.

[9] Tinline R, Rosatte R, MacInnes C (2002) Estimating the incubation period of raccoon rabies: A time–space clustering approach. Preventative Veterinary Medicine 56: 89–103. 10.1016/s0167-5877(02)00126-5 12419602

[10] Childs JE, Curns AT, Dey ME, Real LA, Feinstein L, et al (2000) Predicting the local dynamics of epizootic rabies among raccoons in the United States. Proceedings of the National Academy of Sciences of the United States of America 97: 13666–13671. 11069300 10.1073/pnas.240326697 PMC17633

[11] Hanlon CA, Niezgoda M, Rupprecht CE (2007) Rabies in terrestrial animals. In: Jackson AC, Wunner WH editors. Rabies 2nd edition. London: Academic Press. pp. 201–258.

[12] Kappus KD, Bigler WJ, McLean RG, Trevino HA (1970) The raccoon and emerging rabies host. Journal of Wildlife Diseases 6: 507–509. 16512166 10.7589/0090-3558-6.4.507

[13] Nettles VF, Shaddock JH, Sikes RK, Reyes CR (1979) rabies in translocated raccoons. American Journal of Public Health 69: 601–602. 443502 10.2105/ajph.69.6.601 PMC1618975

[14] Smith JS, Yager PA, Bigler WJ, Hartwig ECJ (1990) Surveillance and epidemiological mapping of monoclonal antibody-defined rabies variants in Florida. Journal of Wildlife Diseases 26: 473–485. 2250324 10.7589/0090-3558-26.4.473

[15] Wilson ML, Bretsky PM, Cooper GH, Egbertson SH, Van Kruiningen HJ, et al (1997) Emergence of raccoon rabies in Connecticut. 1991–1994: Spatial and temporal characteristics of animal infection and human contact. American Journal of Tropical Medicine and Hygiene 57: 457–463. 9347964 10.4269/ajtmh.1997.57.457

[16] Moore DA (1999) Spatial diffusion of raccoons rabies in Pennsylvania, USA. Preventive Veterinary Medicine 40: 19–32. 10343331 10.1016/s0167-5877(99)00005-7

[17] Roscoe DE, Holste WC, Sorhage FE, Campbell C, Niezgoda M, et al (1998) Efficacy of an oral vaccinia-rabies glycoprotein recombinant vaccine in controlling epidemic raccoon rabies in New Jersey. Journal of Wildlife Diseases 34: 752–763. 9813845 10.7589/0090-3558-34.4.752

[18] Rosatte R, Sobey K, Donovan D, Bruce L, Allan M, et al (2006) Behavior, movements, and demographics of rabid raccoons in Ontario, Canada: management implications. Journal of Wildlife Diseases 42: 589–605. 17092890 10.7589/0090-3558-42.3.589

[19] Rosatte RC, Donovan D, Allan M, Bruce L, Buchanan T, et al (2009) The control of raccoon rabies in Ontario Canda: proactive and reactive tactics, 1994–2007. Journal of Wildlife Diseases 45: 772–784. 19617488 10.7589/0090-3558-45.3.772

[20] Cullingham CI, Pond BA, Kyle CJ, Rees EE, Rosatte RC, et al (2008) Combining direct and indirect genetic methods to estimate dispersal for informing wildlife disease management decisions. Molecular Ecology 17: 4874–4886. 19140978 10.1111/j.1365-294X.2008.03956.x

[21] Recueno S, Eidson M, Cherry B, Kulldorff M, Johnson G (2008) Factors associated with endemic raccoon (*Procyon lotor*) rabies in terrestrial mammals in New York State, USA. Preventative Veterinary Medicine 86: 30–42. 10.1016/j.prevetmed.2008.03.001 18406482

[22] Murray, Stanley EA, Brown DL (1986) On the spatial spread of rabies among foxes. Proceedings of the Royal Society of London B 229: 111–150. 10.1098/rspb.1986.0078 2880348

[23] Robert K, Garant G, Pelletier F (2012) Keep in touch: Does spatial overlap correlate with contact rate frequency? The Journal of Wildlife Management DOI: 10.1002/jwmg.435.

[24] Newman MEJ (2002) The spread of epidemic disease on networks. Physical Review E 66: 016128. 10.1103/PhysRevE.66.016128 12241447

[25] Keeling MJ, Eams KTD (2005) Networks and epidemic models. Journal of the Royal Society Interface 2: 295–307. 10.1098/rsif.2005.0051 PMC1578276 16849187

[26] Keeling MJ, Danon L, Vernon MC, House TA (2010) Individual identity and movement networks for disease metapopulations. Proceedings of the National Academy of Sciences 107: 8866–8870. 10.1073/pnas.1000416107 PMC2889353 20421468

[27] Ewer RF (1973) The Carnivores. IthacaNew York: Cornell University Press. 459 p.

[28] Barash DP (1974) Neighbor recognition in two ‘solitary’ carnivores: the raccoon (*Procyon lotor*) and the red fox (*Vulpes fulva*). Science 185: 794–796. 17799055 10.1126/science.185.4153.794

[29] Wilson EO (1975) Sociobiology. Cambridge: Harvard University Press. 697 p.

[30] Kaufmann JH (1982) Raccoon and allies. In: Feldhamer GA, Thompson BC, Chapman JA editors. Wild mammals of North America: biology, management, and economics. Baltimore: John Hopkins University Press. pp 567–585.

[31] Sandell M (1989) The mating tactics and spacing patterns of solitary carnivores. In: Gittleman JL editor. Carnivore behavior, ecology, and evolution. Ithaca: Cornell University Press. pp. 164–182.

[32] Gehrt SD, Fritzell EK (1998) Resource distribution, female home range dispersion and male spatial interactions: group structure in a solitary carnivore. Animal Behaviour 55: 1211–1227. 9632506 10.1006/anbe.1997.0657

[33] Chamberlain MJ, Leopold BD (2002) Spatio-temporal relations among adult raccoons (*Procyon lotor*) in central Mississippi. American Midland Naturalist 148: 297–308.

[34] Gehrt SD, Fox LB (2004) Spatial patterns and dynamic interaction among raccoons in eastern Kansas. Southwestern Naturalist 49: 116–121.

[35] Pitt JA, Lariviere S, Messier F (2008) Social organization and group formation of raccoons at the edge of their distribution. Journal of Mammalogy 89: 646–653.

[36] Hirsch BT, Prange S, Hauver S, Gehrt SD (2013) Genetic relatedness does not predict raccoon social network structure. Animal Behaviour 85: 463–470.

[37] Prange S, Gehrt SD, Wiggers EP (2003) Demographic factors contributing to high raccoon densities in urban landscapes. Journal of Wildlife Management 67: 324–333.

[38] Prange S, Gehrt SD, Wiggers EP (2004) Influences of anthropogenic resources on raccoon (Procyon lotor) movements and spatial distribution. Journal of Mammalogy 85: 483–490.

[39] Prange S, Jordan T, Hunter C, Gehrt SD (2006) New radiocollars for the detection of proximity among individuals. Wildlife Society Bulletin 34: 1333–1344.

[40] Ryder TB, Horton BM, van den Tillart M, Morales JDD, Moore IT (2012) Proximity data-loggers increase the quantity and quality of social network data. Biology Letters. DOI:10.1098/rsbl.2012.0536. 10.1098/rsbl.2012.0536 PMC3497117 22859558

[41] Hamade RK, Bashford J, McCallum HI, Jones ME (2009) Contact networks in a wild Tasmanian devil (*Sarcophilus harrissii*) population: using social network analysis to reveal seasonal variability in social behaviour and its implications for transmission of devil facial tumour disease. Ecology Letters 12: 1147–1157. 19694783 10.1111/j.1461-0248.2009.01370.x

[42] Bohm M, Hutchings MR, White PCL (2009) Contact networks in a wildlife-livestock host community: Identifying high-risk individuals in the transmission of bovine TB among badgers and cattle. PLOS One, 4, e5016. 10.1371/journal.pone.0005016 PMC2660423 19401755

[43] Gehrt SD, Hungerford LL, Hatten S (2001) Drug effects on recaptures of raccoons. Wildlife Society Bulletin 29: 833–837.

[44] Keeling MJ (1999) The effects of local spatial structure on epidemiological invasions. Proceedings of the Royal Society B. 266: 859–867. 10.1098/rspb.1999.0716 PMC1689913 10343409

[45] Grau GA, Sanderson GC, Rogers JP (1970) Age determination in raccoons. Journal of Wildlife Management 34: 364–372.

[46] Hamade RK, Bashford J, Jones ME, McCallum HI (2012) Simulating devil facial tumour disease outbreaks across empirically derived contact networks. Journal of Applied Ecology 49: 447–456.

[47] Beasley JC, Olson ZH, Beatty WS, Dharmarajan G, Rhodes OE Jr (2013) Effects of Culling on Mesopredator Population Dynamics. PLoS ONE 8(3): e58982 doi:10.1371/journal.pone.0058982 23527065 10.1371/journal.pone.0058982 PMC3604110

[48] Grant JWA, Girard IL, Breau C, Weir LK (2002) Influence of food abundance on competitive aggression in juvenile convict cichlids. Animal Behaviour 63: 323–330.

[49] Hirsch BT (2007) Costs and benefits of within-group spatial position: a feeding competition model. Quarterly Review of Biology 82: 9–27. 17354992 10.1086/511657

[50] Seidensticker J, Johnsingh AJT, Ross R, Sanders G, Webb MB (1988) Raccoons and rabies in Appalachian mountain hollows. National Geographic Research 4: 359–370.

[51] Hauver S, Hirsch BT, Prange S, Dubach J, Gehrt SD (2013) Age, but not sex or genetic relatedness, shapes raccoon dominance patterns. Ethology. 119: 769–778.

[52] Hubbard DR (1985) A descriptive epidemiologic study of raccoon rabies in a rural environment. Journal of Wildlife Diseases 21: 105–110. 3999245 10.7589/0090-3558-21.2.105

[53] Jenkins SR, Winkler WG (1987) Descriptive epidemiology from an epizootic of raccoon rabies in the mid-Atlantic states, 1982–1983. American Journal of Epidemiology 126: 429–437. 3497576 10.1093/oxfordjournals.aje.a114674

[54] Winkler WG, Jenkins SR (1991) Raccoon rabies. In: Baer GR editor. The natural history of rabies 2^nd^ edition. Boca Raton: CRC press. pp 325–340.

[55] Bigler WR, McLean RG, Trevino HA (1973) Epizootic aspects of raccoon rabies in Florida. American Journal of Epidemiology 98: 326–335. 4583405 10.1093/oxfordjournals.aje.a121562

[56] Jenkins SR, Perry BD, Winkler WG (1988) Ecology and epidemiology of raccoon rabies. Reviews of Infectious Diseases 10: S620–S625. 3264616 10.1093/clinids/10.supplement_4.s620

[57] Raizman EA, Dharmarajan G, Beasley JC, Wu CC, Pogranichniy RM, et al (2009) Serologic survey for selected infectious diseases in raccoons (*Procyon lotor*) in Indiana, USA. Journal of Wildlife Diseases 45: 531–536. 19395767 10.7589/0090-3558-45.2.531

[58] Dharmarajan G, Beasley JC, Fike JA, Raizman EA, Wu CC, et al (2012) Effects of kin-structure on disease dynamics in raccoons (*Procyon lotor*) inhabiting a fragmented landscape. Basic and Applied Ecology 13: 560–567.

[59] Deem SL, Spelman LH, Yates RA, Montali RJ (2000) Canine distempter in terrestrial carnivores: A review. Journal of Zoo and Wildlife medicine 31: 441–451. 11428391 10.1638/1042-7260(2000)031[0441:CDITCA]2.0.CO;2

[60] Greene GE, Appel MJ (1990) Canine distemper. In: Greene CE editor. Infectious diseases of the dog and cat. Philadelphia: W. B. Saunders. pp 226–241.

[61] Hoff GL, Bigler WJ (1974) Epizootic of canine distemper virus infection among urban raccoons and gray foxes. Journal of Wildlife Diseases 10: 423–428. 4436928 10.7589/0090-3558-10.4.423

[62] Cranfield MR, Barker IK, Mehren KG, Rapley WA (1984) Canine distemper in wild raccoons (Procyon lotor) at the Metropolitan Toronto Zoo. Canadian Veterinary Journal 25: 63–66. 17422359 PMC1790529

[63] Roscoe DE (1993) Epizootiology of canine distemper in New Jersey raccoons. Journal of Wildlife Diseases 29: 390– 395. 10.7589/0090-3558-29.3.390 8355340

[64] Mitchell MA, Hungerford LL, Nixon C, Esker T, Sullivan J, et al.. (1997) Serologic survey for selected infectious disease agents in raccoons from Illinois. Journal of Wildlife Diseases 35: 347– 355. 10.7589/0090-3558-35.2.347 10231761

[65] Wright AN, Gompper ME (2005) Altered parasite assemblages in raccoons in response to manipulated resource availability. Oecologia 144: 147–155. 10.1007/s00442-005-0018-3 15891856

